# In Utero Extrahepatic Bile Duct Damage and Repair: Implications for Biliary Atresia

**DOI:** 10.1177/10935266241247479

**Published:** 2024-05-19

**Authors:** Iris E. M. de Jong, Rebecca G. Wells

**Affiliations:** 1 Department of Medicine, University of Pennsylvania, Philadelphia, PA, USA; 2 Center for Engineering MechanoBiology, University of Pennsylvania, Philadelphia, PA, USA; 3 Department of Pathology and Laboratory Medicine, University of Pennsylvania, Philadelphia, PA, USA; 4 Department of Bioengineering, University of Pennsylvania, Philadelphia, PA, USA

**Keywords:** redox stress, peribiliary gland, bile duct development, fetal wound healing, hyaluronic acid

## Abstract

Biliary atresia (BA) is a cholangiopathy affecting the extrahepatic bile duct (EHBD) of newborns. The etiology and pathophysiology of BA are not fully understood; however, multiple causes of damage and obstruction of the neonatal EHBD have been identified. Initial damage to the EHBD likely occurs before birth. We discuss how different developmental stages in utero and birth itself could influence the susceptibility of the fetal EHBD to damage and a damaging wound-healing response. We propose that a damage-repair response of the fetal and neonatal EHBD involving redox stress and a program of fetal wound healing could—regardless of the cause of the initial damage—lead to either obstruction and BA or repair of the duct and recovery. This overarching concept should guide future research targeted toward identification of factors that contribute to recovery as opposed to progression of injury and fibrosis. Viewing BA through the lens of an in utero damage-repair response could open up new avenues for research and suggests exciting new therapeutic targets.

## Introduction

Biliary atresia (BA) is a disease of newborns characterized by fibrosis that primarily targets the extrahepatic bile ducts (EHBD). Neonates who go on to develop BA universally display biochemical abnormalities, although they appear healthy at birth; within a few weeks, however, they develop clinical symptoms including jaundice, pale stool, dark urine, abdominal pain, and high direct bilirubin levels, consistent with an atretic and obstructed EHBD. The current standard of care is for patients to undergo a palliative surgical biliary drainage procedure, known as a Kasai hepatoportoenterostomy, shortly after diagnosis. Drainage is successful in about half, but all patients will ultimately develop liver fibrosis and progress to end-stage liver disease. Approximately 50% will require a liver transplant before the age of 2 years, and nearly all before reaching adulthood.^1^

The development of alternative and potentially curative therapies for BA has been hampered by delays in diagnosis (which often occurs several months after the onset of symptoms, when irreversible damage has occurred) and by a lack of understanding of disease pathogenesis. New approaches to screening have led to significantly earlier diagnosis, with improved outcomes,^2^ although the bile duct remnants obtained even at early Kasai surgeries demonstrate scarring and often lack a patent lumen, with only rare and metaplastic epithelial structures.^3^ Nonetheless, it is possible that still earlier diagnosis and intervention would offer the possibility of duct repair as opposed to progressive fibrosis.

BA can occur as an isolated condition (80–85%) or in combination with laterality defects or other major malformations (syndromic BA, 15–20%); the etiopathogenesis of the 2 variants, while unknown for both, may differ.^4^ Compelling evidence, in particular the *universal* finding of elevated direct/conjugated bilirubin when measured within 72 hours of birth in babies who go on to develop BA, suggests that the injury underlying BA occurs during gestation.^2^ Multiple insults that primarily or exclusively affect the EHBD have been proposed, including viral infections, autoimmune diseases, and environmental toxins (e.g., agents similar to biliatresone). These are likely impacted by factors including the fetal and neonatal immune response, genetic modifiers, and developmental immaturity of the EHBD (e.g., a leaky epithelium, patchy epithelial glycocalyx, and sparse submucosal barrier) that increase susceptibility of the fetal/neonatal duct to injury.^5-10^ Regardless of the etiology of BA, which may be multifactorial and different in each patient, understanding the general response of the fetal bile duct to an insult is critical to understanding the pathophysiology of BA. Unfortunately, it is difficult to obtain well-preserved tissue from healthy human fetuses, and, given that the diagnosis is made after birth, effectively impossible to obtain tissue or imaging information from BA-affected fetuses. Additionally, animal models fail to capture the complexity of the disease. The most widely used animal model of BA is the rhesus rotavirus (RRV) model, whereby BALB/c mouse pups are infected with RRV within 48 hours (ideally 24 hours) of birth. Since bile duct damage is initiated postnatally, this model does not incorporate fetal developmental stages, transitions between fetal and adult wound healing programs, or the unique fetal environment that includes the fetal vascular system, immature immune and endocrine systems, cellular metabolism, and relative hypoxemia. Equally if not more important, it does not encompass birth-associated stress.

This review does not discuss further the potential etiologies of BA, which are well covered in other reviews,^4,11-14^ but rather describes injury susceptibility and the damage-repair response of the fetal EHBD, then outlines a possible scenario by which progressive biliary injury could occur. The review ends by highlighting critical new areas for investigation.

## Development of the Large Bile Ducts

### Basic Bile Duct Anatomy

**Figure 1.**
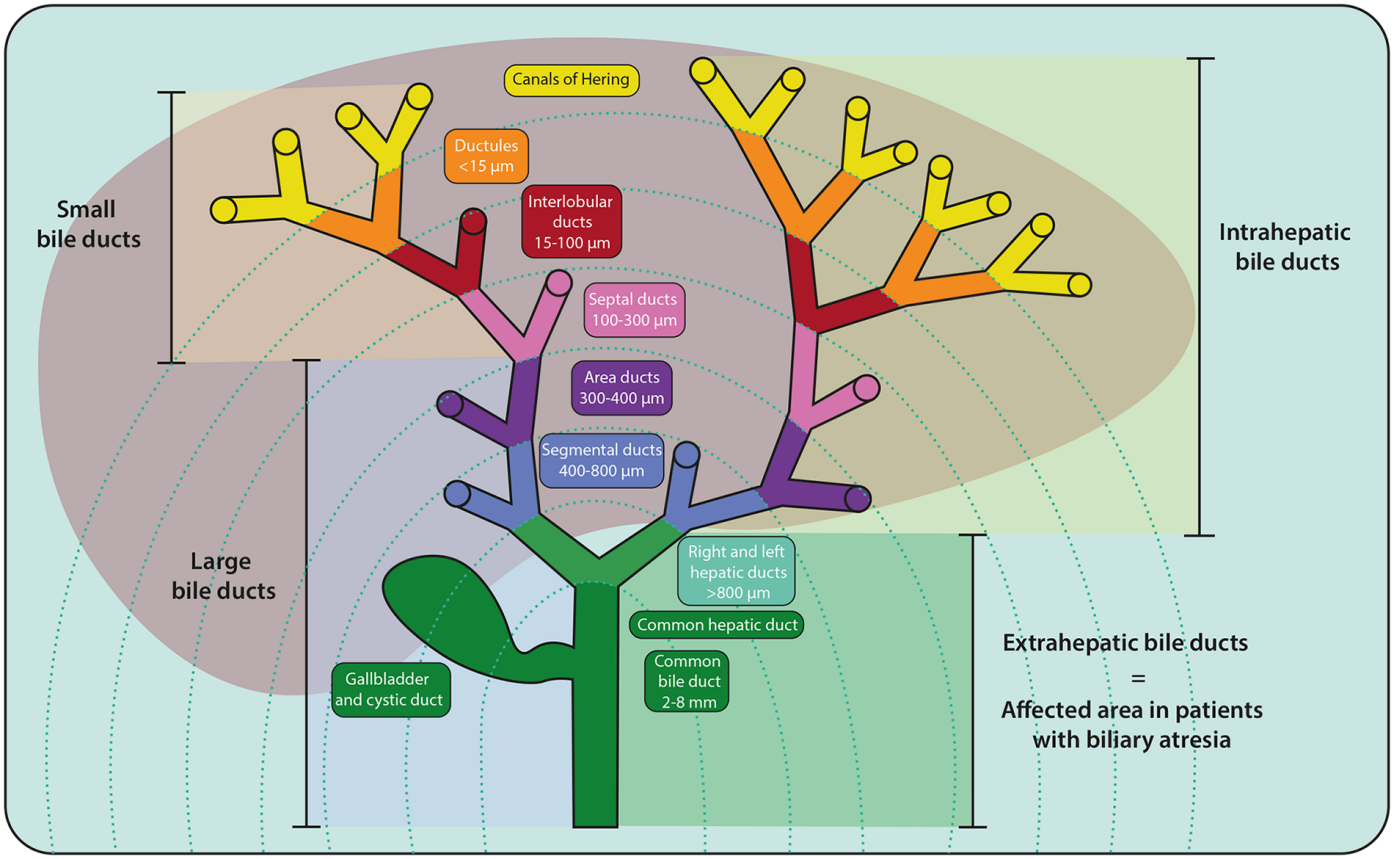
Nomenclature of the biliary tree. An arborizing network of tubular ducts spans from the periphery of the liver to the hilum, merging into the common bile duct, which drains into the duodenum. The smallest diameter ducts, the canals of Hering, are aligned with hepatocytes and lead progressively to the ductules, interlobular ducts, septal ducts, area ducts, segmental ducts, hepatic ducts, and common bile duct. Different order ducts are categorized based on their location (intra vs extra) or size and phenotype (small vs large).

The adult biliary tree consists of an arborizing network with multiple branching orders (Figure 1). Bile is produced by hepatocytes and collected in bile canaliculi (which are formed by the apical domains of hepatocytes), then flows into the canals of Hering, which are lined by both hepatocytes and cholangiocytes. Bile then flows through the ductules into the interlobular ducts, the septal ducts, area ducts, segmental ducts, hepatic ducts and, eventually, into the common hepatic duct and common bile duct (Figure 1). Within the liver, the diameter of the ducts progressively increases from the periphery of the liver toward the hepatic hilum, defined as the opening of the liver through which the hepatic ducts and vascular structures descend. The terminology describing groups of ducts can be confusing. The extrahepatic portion of the right and left hepatic ducts, the common hepatic duct, cystic duct, and common bile duct are located outside the liver and are collectively referred to as the EHBD. This is distinct from the classification of large vs. small ducts: the area ducts through the common bile duct are defined as the large bile ducts while the canals of Hering, ductules, interlobular ducts, and septal ducts are defined as the small bile ducts (Figure 1). Small ducts are lined by cuboidal small cholangiocytes and the large ducts by columnar large cholangiocytes. These cell types have distinct behaviors in response to stimuli.^15-18^ The total number of biliary branching orders ranges between 7 and 11, depending on the method of classification.^19,20^

Peribiliary glands (PBGs) are glands derived from the main ducts that protrude in a monopodial growth pattern into the mesenchyme surrounding the biliary tree.^16^ They are secretory glands (primarily secreting mucus) that also harbor cholangiocyte progenitors, which migrate to repair lost surface epithelial cells after severe damage.^21-23^ PBG progenitor cells also contribute to the homeostatic turnover of the epithelium in a compartmentalized fashion.^24^ In humans, PBGs take many shapes and are classified as either simple, coiled, or branched tubular; simple or branched alveolar; or compound tubular, alveolar, or tubuloalveolar.^25,26^ Simple tubular structures are generally located near the lumen and are called *periluminal PBGs*, whereas tubuloalveolar structures are located deeper in the bile duct wall and are called *deep PBGs*. These terms should be chosen over *intra-and extramural PBGs* as the border between intra-and extramural is not always clear. In certain cases, the distinction between periluminal and deep PBGs can be challenging given that tubuloalveolar structures occasionally appear close to the lumen (note the structural heterogeneity in 3D corrosion casts in ref. Kordzaia et al^26^). Then, cell morphology (cuboidal cells), phenotype (Muc6+/Sox9+ /Aqp5+/Olfm4+/Trop2−) and structure (small, clustered acini) could identify tubuloalveolar deep PBGs from simple tubular periluminal PBGs in 2D sections.^26^ Moreover, PBG structures occasionally connect different parts of the ducts, creating what are essentially ductular collaterals.^26^ PBGs are predominately associated with the large bile ducts, with sparse PBGs around the septal ducts, and few to none around the smaller ducts.^27^ Between the segmental ducts and the smallest ducts that have PBGs, PBG orifices appear in longitudinal opposing lines, whereas the PBGs surrounding the larger ducts arise from the surface epithelium in random directions.^26^

### The Timing of Human Bile Duct Development

**Figure 2.**
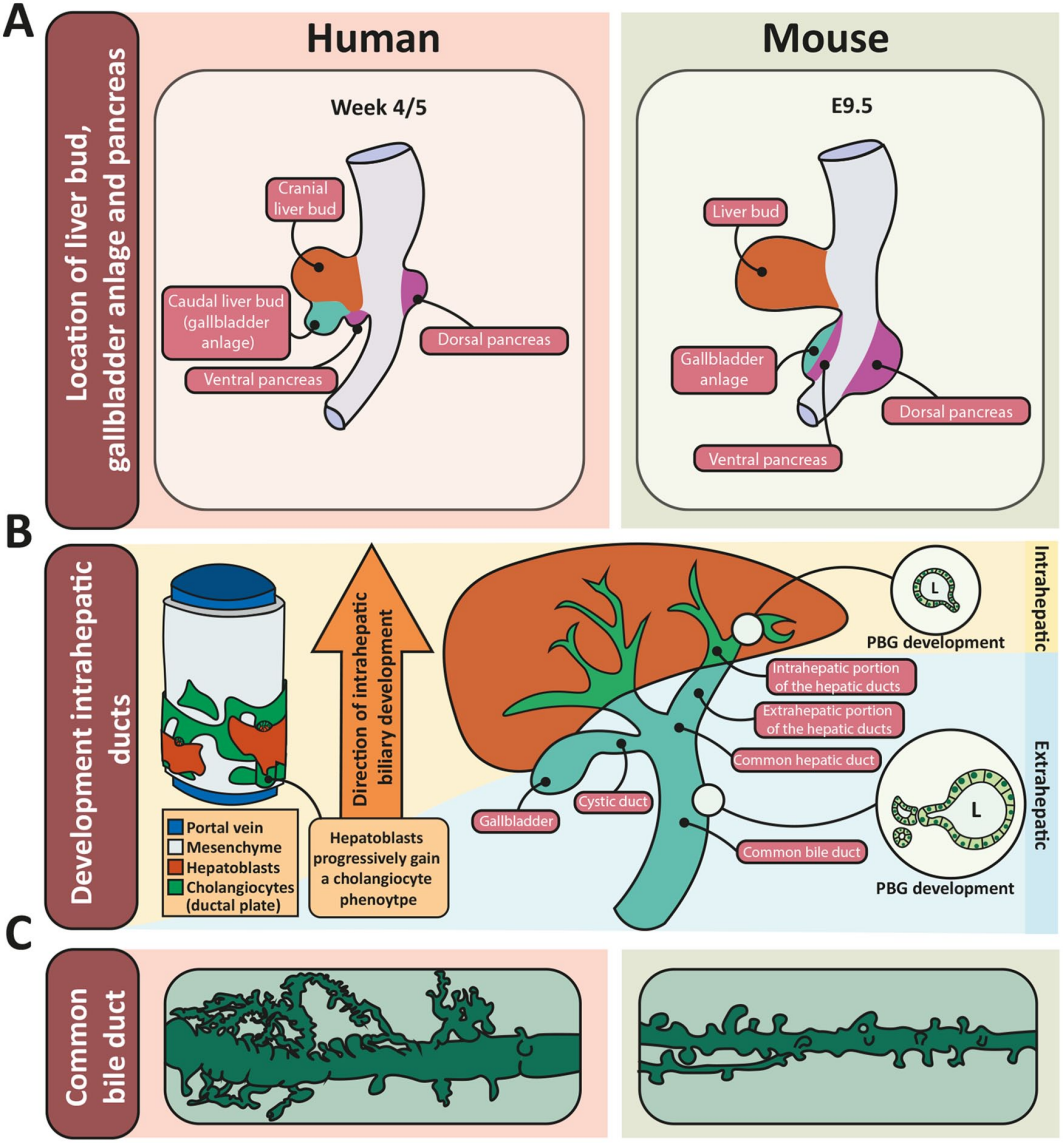
Development of the biliary tree and morphology of PBGs in human and mouse. (A) Location of the liver bud, gallbladder anlage, and pancreas in early development in human (left) and mouse (right). Based on Figure 3 in Lemaigre.^31^ (B) Development of the intrahepatic bile ducts from the ductal plate (in green, left) and development of intra- and extrahepatic PBGs (circular insets on right). Lumens are formed between the hepatoblast and cholangiocyte layer yielding tubes aligned with the portal vein. After formation of a lumen, the hepatoblasts differentiate to a biliary phenotype. This is a progressive process from the hilum toward the periphery of the liver (left). The intrahepatic PBGs start to bud off from the epithelium after the mature duct is formed, following the direction of the formation of the intrahepatic ducts, while at that timepoint (10/13 weeks of gestation), the EHBD is complete and starts to form PBGs at all levels (right). (C) The PBG network surrounding the large bile ducts in human (left) and mouse (right). Note the increased branching and complexity in the human PBG network. Drawn based on underlying information in published work. 157, 38, 26

**Figure 3.**
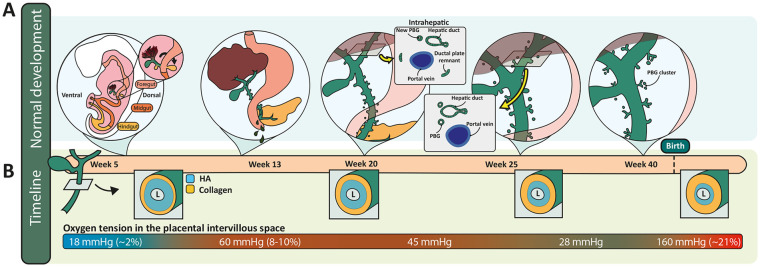
Normal development of the EHBDs in human. (A) Normal development of the EHBDs including a cross-section of the removal of the ductal plate after formation of the intrahepatically-located hepatic ducts at the level of the hilum (insets). The corresponding timepoints in gestation are indicated in (B) with an estimate of the spatiotemporal changes in HA and collagen deposition in the duct submucosa, based on ref. de Jong et al.^32^ Oxygen tension in the intervillous space of the placenta (bottom of B) is used to estimate the oxygen supply to the fetus (which has not been measured directly). Abbreviations: HA, hyaluronic acid; PBG, peribiliary gland.

It is critical to understand bile duct development and appreciate the differences in timing between humans and mice in order to study the pathophysiology of BA and interpret the results from animal models. The EHBD develops from the liver bud, which arises ventrally from the foregut endoderm (Figures 2(a) and 3(a)). The liver bud undergoes caudal elongation, rapidly forming a (patent) tube, called the caudal bud or pars cystica, from which the gallbladder, cystic duct, and common bile duct develop. At around 5 weeks of gestation in humans, the gallbladder anlage becomes evident and the EHBD and the ventral pancreatic bud rotate to their definitive positions at the dorsal side of the duodenum, where the ventral and dorsal pancreas merge. In contrast to the extrahepatic component, the intrahepatic portion of the large duct system as well as the small duct system develop from the ductal plate, a discontinuous sleeve of cholangiocytes (Figure 2(b), left) that first appears around gestational week 7/8 at the hilum and develops progressively thereafter toward the periphery of the liver bud. Lumens form between the cholangiocytes and adjacent hepatoblasts, making tubes that are aligned with the portal vein. The hepatoblasts progressively adopt a biliary phenotype (Figure 2(b), left)^28,29^; this process continues until 1 month after birth.^30^

The current understanding is that the intrahepatic bile ducts develop from the ductal plate, and therefore from a different anlage (cranial liver bud) than the extrahepatic ducts (caudal liver bud/gallbladder anlage) (Figure 2(a)). In this context, it is important to note that discontinuity between the 2 duct systems has never been observed, and it is yet unclear how they connect during development.^30,31^

The development of ductular structures at the level of the hilum occurs between gestational weeks 10 and 13 in humans and, given that hilar duct abnormalities are typical of BA, provides important context for understanding the pathogenesis of this disease. The extrahepatic portions of the left and right hepatic ducts, which develop from the common hepatic duct at 11/12 weeks of gestation,^30,33^ remain continuous with the common hepatic duct and connect proximal to the hilum to several primitive ductular structures that emerge from the ductal plate.^31^ Within the liver, these primitive ductular structures interconnect and elongate to form the intrahepatic portions of the hepatic ducts.^30,33,34^ During development of the intrahepatic ducts, the surrounding mesenchyme condenses and increases in thickness. Eosinophils, neutrophils and mononuclear cells are found around the remodeling ducts and disappear after completion of remodeling, which occurs by 12/13 weeks of gestation.^33^ By week 25, all residual ductal plate remnants at the hilum are gone (Figures 2(b) and 3(a)).^33^

After the right and left hepatic ducts are formed, around gestational week 12, bile secretion begins, albeit at low levels compared to after birth. PBGs begin to sprout from the epithelial lining of the common bile duct around weeks 10/11 (Figure 3(a)), and mucus from the PBGs is added to the secreted bile. PBG mucus production and bile secretion increase as the time of birth approaches.^35^

The mechanism of development of the intrahepatic PBGs is likely comparable to that of the PBGs lining the extrahepatic ducts, based on similar 3D structures of the PBG network in adulthood.^26^ PBG development occurs at all levels in a monopodial growth pattern, meaning that lateral branches (the glands) develop from the main duct. However, the common bile duct forms well before the intrahepatic ducts, resulting in delayed PBG development in the intrahepatic ducts compared to the extrahepatic ducts.^16,27^

Extrahepatic PBGs develop first via budding off the surface epithelium of the common bile duct, initially encircling and evenly distributed throughout the bile duct wall. They increase in number, reaching their peak around birth. Extrahepatic PBG density, however, reaches a peak directly after formation and progressively decreases by virtue of the rapidly increasing bile duct diameter, which plateaus at the age of 1 year.^36^ Toward birth, starting from gestational week 20, the PBGs of all large bile ducts start to aggregate in clusters as is seen in adult large bile ducts (Figures 2(c) and 3(a)).^34^

PBGs around the intrahepatic portion of the (large) hepatic ducts have been observed as early as 10 weeks of gestation and, in contrast to the PBGs of the extrahepatic ducts, steadily increase in number and density over time until age 15 years.^27^ The different patterns of PBG density between extra- and intrahepatic ducts can be explained by the developmental patterns of the 2 duct systems: the surface epithelia of the extrahepatic ducts have a biliary phenotype from the start and the ducts increase progressively in diameter during development, resulting in early PBG budding and decreasing PBG density over time. In contrast, the intrahepatic PBGs bud off the surface epithelium after the epithelium has differentiated to a biliary phenotype and after a lumen has formed. At this stage, the diameter of the ducts does not increase as much as occurs for the extrahepatic portion, explaining an increasing PBG density over time. In line with the different time courses of intra- and extrahepatic large duct development, the extrahepatic ducts show low rates of proliferation compared to their intrahepatic counterparts during the remodeling phase of the intrahepatic ducts (i.e., gestational weeks 10–13).^30,35^

### Differences in Bile Duct Development Between Mice and Humans

There are significant differences between mouse and human in timing of development, anatomy, and physiology of the biliary tree; these impact the interpretation of results from animal models of BA.

In general, organ development can vary between animal species in duration, rate, and order. A shift in timing (called heterochrony) is common between species and can occur both between organs and within a specific organ.^37-39^ Heterochronies between human and mouse biliary developmental programs are not fully described and should be investigated separately for each level of ducts. Regardless, the overall prenatal development of the biliary tree in mice resembles that in humans except for the origin of the extrahepatic portion of the biliary tree. In humans, the caudal part of the foregut-derived liver bud develops into the extrahepatic bile ducts and gallbladder, whereas in mice a separate foregut-derived diverticulum, caudal to the liver bud, gives rise to the extrahepatic bile ducts, gallbladder, and ventral pancreas (Figure 2(a)).^31^ Additionally, the ductal plate emerges around E13.5 (past the midpoint of gestation) in mice and around gestational week 7/8 in humans. We found that the extracellular matrix (ECM) of the EHBD in postnatal mice and rats is similar to the EHBD of fetal sheep and humans, suggesting delayed deposition of biliary ECM components in rodents compared to humans.^32^

While the details of PBG network development in mice are not known, the architecture of the adult network clearly differs between mouse and human (Figure 2(c)). In contrast to the complex and branched PBG architecture in humans, the PBG network of adult mice contains predominantly single tubular-shaped PBGs, equally dispersed, occasionally with a tubule running parallel to the lumen and connecting acini in different parts of the wall.^40^

Figure 3(a) is a schematic of the progression of human bile duct structure through consecutive developmental stages. In the next section, we discuss the damage-repair response of the fetal bile ducts and how it is influenced by developmental stages and conditions in utero.

## In Utero Damage

The uterine environment in which the fetus resides is, by definition, different than the postnatal environment. How this environment impacts susceptibility of the fetal EHBD to damage is unexplored, but studies in other organ systems may provide important clues. Insults that occur during periods of organ and tissue development can lead to dysmorphogenesis, which is different than adult responses to injury. In this section we discuss the influence of the uterine environment and birth on bile duct susceptibility to damage, particularly in the context of fetal development and anatomy.

### Redox Environment During Development

**Figure 4.**
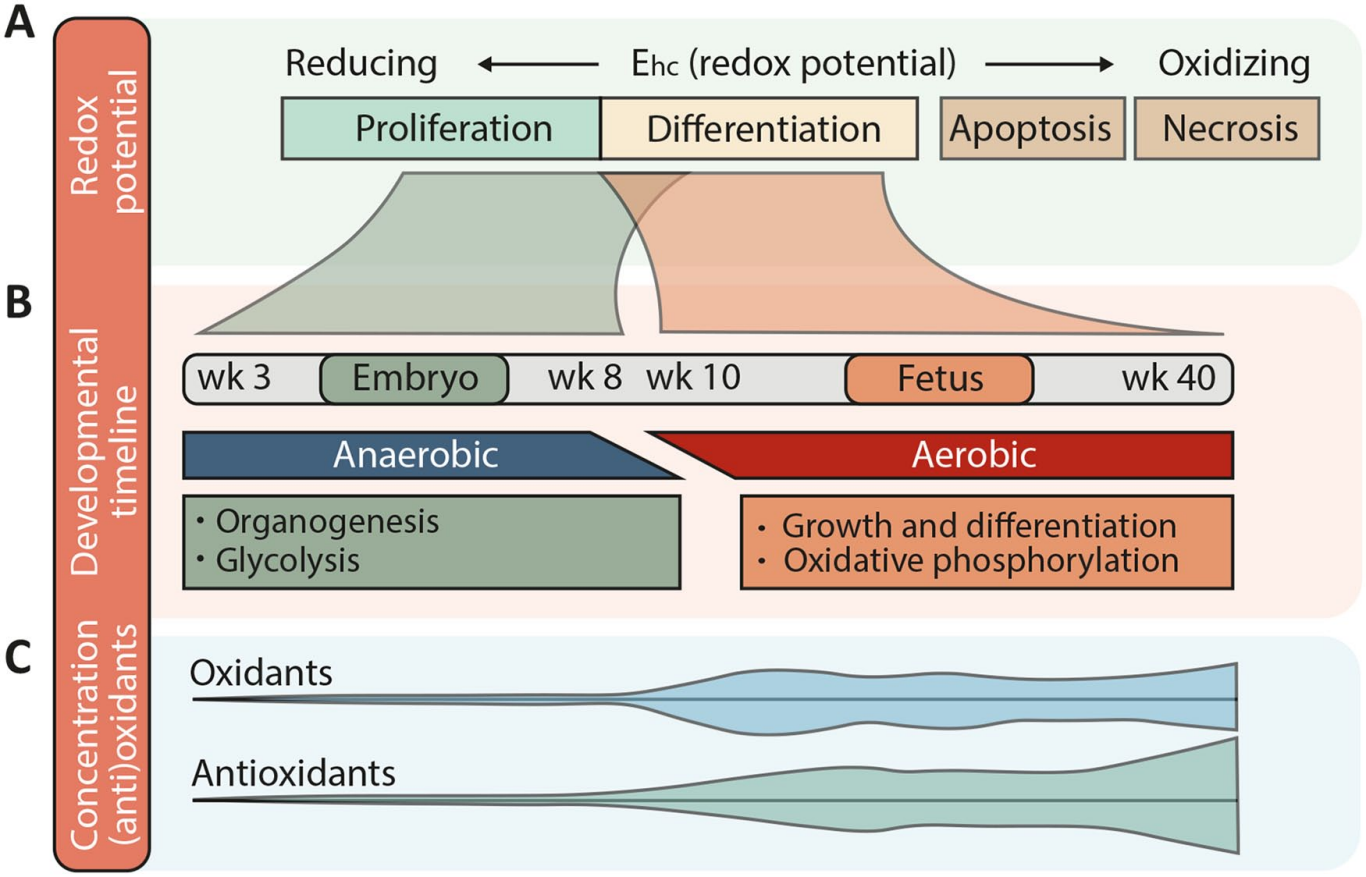
Redox environment during gestation in humans. (A) When the redox potential (Ehc) is low, the redox environment is reducing, promoting cellular proliferation, while a higher redox potential and therefore a more oxidizing milieu promotes differentiation or even apoptosis and necrosis. (B) A gestational timeline showing periods when the environment is reducing (embryo, GA week 3–8) or slightly more oxidized (fetus, GA week 10–40). These 2 periods during human gestation are associated with anaerobic vs aerobic metabolism and different morphological and functional programs of development. Compare these changes also with the oxygen levels displayed in Figure 3(b). (C) Graphical representation of levels of oxidants and antioxidants during human gestation, corresponding to the timeline in (B). This figure is based on refs. Schafer and Buettner,^43^ Hansen,^44^ Dennery,^45^ Hansen and Harris,^46^ Davis and Welty,^47^ and Berkelhamer and Farrow.^48^ Abbreviation: wk, week.

The uterine environment is overall very low in oxygen, although oxygen levels are highly variable and dynamic during gestation (Figure 3(b), bottom). This has a major impact on the growth and development of the embryo (to gestational week 9) and fetus (gestational week 9 until term). Oxygen tension in utero through week 10 is low enough that cellular metabolism primarily takes the form of glycolysis, as opposed to oxidative phosphorylation, which predominates later in gestation.^41,42^ (Figure 4). Oxygen tension increases toward term, although undergoes its most significant increase in the immediate postnatal period, when the lungs expand and start to exchange oxygen, leading to normoxia with an oxygen tension of 160 mmHg.

With increased oxygenation and correspondingly increased mitochondrial adenosine triphosphate (ATP) production, the generation of reactive oxygen species (ROS) after week 10 and as gestation progresses is inevitable. During oxidative phosphorylation, electrons leak from the mitochondrial electron transport chain and react with oxygen, resulting in the formation of the ROS superoxide anion (O_2_−), which is quickly converted by the antioxidant superoxide dismutase (SOD) into oxygen and hydrogen peroxide (H_2_O_2_). Hydrogen peroxide, also a ROS, plays a key role in many redox-sensitive signaling pathways and can be reduced to H_2_O by the enzyme catalase and multiple redox couples. The redox environment in a cell or tissue is calculated by measuring the redox potential (*E*_hc_; expressed in voltage) and the reducing capacity (expressed as concentration of the reduced species in a redox couple) of all redox couples together. This tells us about the buffering capacity of a certain cell or tissue; that is, how easily a tissue can handle increased levels of ROS while avoiding oxidative stress. The redox couple glutathione/disulfide-glutathione (GSSG/2GSH) is both the most extensively studied and the most abundant redox couple in a cell, and therefore the redox state of GSSG/2GSH can be used as an indicator of the cellular redox environment.^43^

During the first 8 weeks of human gestation virtually no ROS are generated, and the redox potential is reducing (GSH *E*_hc_: −250 mV) (Figure 4(a)). Expression of antioxidant enzymes (SOD, glutathione peroxidase, and catalase) is low during this period, consistent with minimal generation of oxidants (Figure 4(c)). The reducing environment promotes proliferation and supports organogenesis in early gestation (Figure 4(b))^43,44^; moreover, the hypoxia promotes maintenance of a stem/progenitor cell phenotype, preventing cholangiocyte differentiation,^49,50^ and stimulates angiogenesis through HIF1, resulting in the expansion of placental vasculature. This, in turn, allows for increased placental oxygen diffusion and a threefold increase in oxygen tension in the placenta by gestational week 10.^51^ Oxygen delivery to the human fetus (as opposed to the placental intervillous space) has proven difficult to measure, but the rise in oxygen tension in the fetus during this period is probably slightly more gradual than in the intervillous space, as depicted in Figure 3(b), providing time for the fetus to adapt.^52^

Of note, oxygen tension in the placental intervillous space surges to 60 mm Hg (8–10% O_2_) around week 10. This oxygen concentration, as compared to extreme hypoxia (1% O_2_) and normoxia (21% O_2_), promoted hepatic stem cell differentiation in an in vitro system into a biliary rather than hepatocyte phenotype.^49^ Although never tested directly in vitro or in vivo, the intermediate oxygen tension between gestational weeks 10 and 13 may contribute to the formation of the ductal plate and a progressive biliary remodeling phase at the level of the hilum.

When the transition to aerobic metabolism takes place at the end of the first trimester, ROS levels increase and the redox potential becomes slightly more oxidized (GSH E_hc_: -220 mV) (Figure 4(a)), stimulating differentiation of fetal tissues.^45,46^ ROS levels increase, with concomitant increases in expression of antioxidant enzymes to maintain the redox balance. Cells that are unable to self-regulate this way become increasingly oxidized and eventually undergo apoptosis (GSH *E*_hc_: less than −190 mV) or, if the oxidative stress is severe, necrosis (Figure 4(a)). The fetal transition to oxidative metabolism is a time of increased risk for miscarriage—the ROS burst and increased oxidative stress at the end of the first trimester can have deleterious effects if the placenta is not properly developed.^53^

### Oxidative Stress and Biliary Atresia

Oxidative stress has been implicated in the pathophysiology of BA. Genes involved in glutathione metabolism and other oxidative stress-related processes are upregulated in samples obtained from BA patients^54,55^ and biliatresone, a toxin that is likely the cause of a BA-like disease in Australian livestock, causes biliary damage and obstruction through depletion of GSH and increased oxidative stress in models of zebrafish and mice.^7,56-58^ *N*-acetylcysteine (NAC), a glutathione precursor, replenishes intracellular GSH levels and functions directly as an antioxidant by neutralizing superoxide anions and hydroxyl radicals^59^—it was shown to prevent biliatresone-induced polarity and permeability defects in cholangiocyte spheroids,^7^ emphasizing oxidative stress as an underlying mechanism in EHBD damage. Recently, we identified microcystin-RR (MC-RR), a product of blue-green algae, as a toxin specific for neonatal mouse and rat cholangiocytes,^60^ and found that MC-RR caused increased ROS levels as well as cholangiocyte monolayer damage in EHBD explants. NAC abrogated the phenotype only when MC-RR doses at the lower end of the active range were administered, raising the possibility that other (non-GSSG/GSH) redox pairs are also important in the EHBD.^60^

Cholangiocytes lining the extrahepatic bile ducts are oxidized at baseline compared to their intrahepatic counterparts.^7,57,61^ In addition, neonatal EHBDs had lower levels of GSH as well as other important antioxidant enzymes when compared to adult ducts, suggesting that the fetal/neonatal EHBD is predisposed to redox injury.^57,58,62^

Oxidative stress may be a common mechanism in BA and EHBD injury from multiple etiologies.^7,44,45,46,56-58^ Similar to biliatresone and MC-RR, viruses modulate cellular redox status during infection.^63^ Cytomegalovirus, which has been associated with BA,^9,64^ increases ROS levels during the early phases of infection after which, at least in adults, glutathione levels increase, enabling avoidance of severe oxidative stress and cell death.^65^ NAC prevents biliary obstruction and increases overall survival in RRV-infected BALB/c pups, suggesting that oxidative stress plays a role in duct damage after RRV infection as well.^55,66^ A small clinical study showed that NAC administration post-Kasai surgery resulted in decreased serum bilirubin, increased bile flow, and an improved intrahepatic immune landscape in BA patients.^67^ A large clinical trial measuring the efficacy of NAC in BA patients is underway.^68^

The association between BA, maternal diabetes, and preterm birth is also consistent with the involvement of redox dysregulation in the pathophysiology of BA.^69^ Gestational diabetes, especially when poorly controlled, leads to increased oxidative stress in the fetus, and it is associated with (besides BA) a myriad of congenital malformations that originate from damage during organogenesis (before gestational week 7), including duodenal atresia, anorectal atresia, situs inversus, and cardiovascular and neural tube defects.^46,52,70-72^ In addition, the association between BA and preterm birth is interesting because antioxidant systems are immature before term (Figure 4(c)), suggesting decreased protection from the oxidative stress typically associated with birth (see below).^73,74^

### Is There a Redox-Related Window of Susceptibility for Fetal EHBD Damage?

The fetal EHBD differs significantly architecturally from the adult, suggesting that an insult during gestation may yield different results than a similar insult during the postnatal period or adulthood. In contrast to the fully developed and collagen-rich adult duct submucosa, the submucosa of fetal bile ducts is largely composed of hyaluronic acid (HA) with little collagen type 1, enabling substances like bile to diffuse through the space easily (Figure 3(b)).^5,32^ The biliary epithelium is different in the fetus as well: cholangiocytes are immature and leaky while PBGs, which function in the adult as a reparative compartment, do not start to protrude into the ductal mesenchyme until gestational week 10 (Figure 3(a)).^27,32^ Moreover, immature postnatal mouse ducts lack a protective glycocalyx, which renders them susceptible to injury through a compromised bicarbonate umbrella.^5,75,76^ Bile salts, particularly hydrophobic bile salts, are damaging to cholangiocytes.^77^ The glycocalyx contributes to a chemical and physical barrier against toxic bile salts by “trapping” bicarbonate close to the apical membrane, ensuring a pericellular alkaline pH, and repulsion of negative deprotonated bile salts.^76^ The lack of a glycocalyx in neonates therefore suggests that they have a decreased tolerance for hydrophobic bile salts and may be at high risk for progressive damage after disruption of the cholangiocyte monolayer.

The period of organogenesis in the embryo (between gestational weeks 3 and 8) is a unique window of susceptibility to exogenous oxidants because antioxidant enzymes are expressed at very low levels (Figure 4(c)).^44,45,46^ Studies with known teratogens that act via redox dysregulation, however, have shown that the same oxidative insult may lead to diverse outcomes depending on the gestational age and tissue due to zonal differences in redox potential, redox couples, and antioxidant enzyme capacities that change over the course of gestation; additionally, certain teratogens affect only specific redox couples, altering some but not all redox-sensitive developmental programs.^46^ Times when the EHBD is specifically sensitive to redox stress have not been identified. To efficiently generate a BA-phenotype in BALB/c mouse pups, RRV inoculation and biliatresone injection must be performed within 48 hours after birth.^12,78,79^ and in zebrafish, susceptibility to biliatresone-induced injury has been shown between 8- and 13-days post fertilization.^80^ In the fetal sheep, we observed significant EHBD damage in response to hypoxia between gestational days 107–128 (mid second trimester), past the period of organogenesis.^32^ Translating these windows of susceptibility to humans may be challenging, if even possible, as there are significant interspecies differences in the response to various oxidative agents.^46^ Moreover, some animals are resistant to specific oxidants throughout gestation.^46^

Taken together, however, these data support the hypothesis that redox dysregulation plays a role in the pathophysiology of BA. Both environmental and viral insults are potential sources of redox changes, leading to shifts in redox potential during gestation and induction of specific redox-sensitive downstream pathways depending on the gestational age at the time of the initial insult. A shift toward a more oxidized redox potential may not lead to extensive necrosis during gestation, but may render the ducts susceptible to a second oxidative hit at the time of birth.

### Fetal Vasculature, the Stress of Birth, and Ischemia-Reperfusion

Birth is a redox stress-intensive event. The fetus experiences multiple ischemia-reperfusion episodes during labor: each uterine contraction reduces uteroplacental perfusion (causing hypoxia), which resumes after uterine relaxation (leading to reperfusion).^81,82^ This causes an overwhelming generation of ROS, although it is normally well-tolerated by the neonate owing to increased antioxidant enzyme expression near term and to extra antioxidants supplied from the maternal side of the placenta (Figure 4(c)).^81,82^

**Figure 5.**
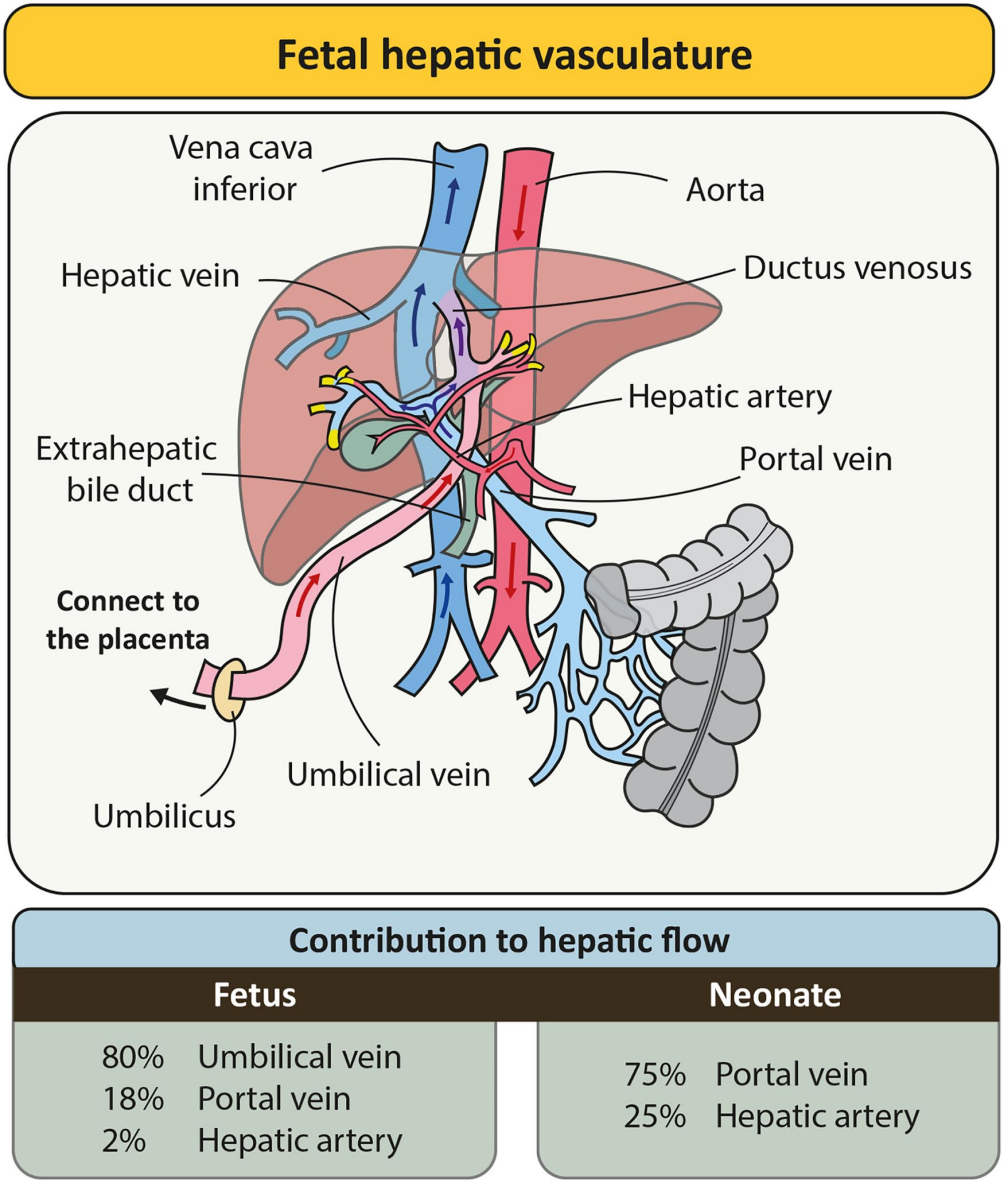
Fetal hepatic vasculature. Graphical representation of the fetal hepatic vasculature showing blood supply to the liver through the umbilical vein (partly shunted), portal vein, and hepatic artery. The relative contribution of each vessel is displayed in the table below for the fetus (left) and neonate/adult (right). The yellow ends of the vessels indicate points where blood supply to the liver occurs.

The EHBD also undergoes ischemia-reperfusion due to the specialized vasculature of the fetus, which secures the oxygen supply to the brain and heart and bypasses (partly) the lungs and liver (Figure 5). Blood to the fetal liver is supplied through the umbilical vein (~80% of total hepatic blood flow) and is partly shunted through the ductus venosus to the inferior vena cava, the hepatic artery (~2% of total hepatic flow) and the portal vein (~18% of total hepatic flow).^83^ Oxygen supply to the liver is heterogeneous, as blood from the portal vein (~30% saturation) enters only the right lobe, and highly oxygenized blood from the umbilical vein (80–85% saturation) primarily supplies the left lobe, although much of it is shunted through the ductus venosus, especially during periods of hypoxia.^83^ After birth, cessation of blood flow through the umbilical vein causes a temporary decrease in total hepatic flow followed by a compensatory increase from both the portal vein and hepatic artery. At the same time, the oxygen content of arterial blood rises as the lungs begin to exchange oxygen. For the hepatic artery this means an increase in oxygen saturation of 40% (from 55% to 95%) and an increase in flow of 10 mL/minute per 100 g liver.^83,84^ The EHBD is almost exclusively nourished by the hepatic artery and thus, these changes during and directly after birth likely lead to a marked increase in oxygen supply to the large bile ducts; this follows a period of relative hypoxemia, and is, by definition, ischemia and reperfusion.

Following ischemia and subsequent reperfusion during adult liver transplantation, bile composition is altered, contributing to bile duct damage.^85^ Bile production is highly adenosine triphosphate (ATP)-dependent,^86,87^ such that after reperfusion immediately post-transplant, the ATP-dependent transporters BSEP (secretion of bile salts) and MDR3 (secretion of phospholipids) have markedly decreased function and only gradually recover, consistent with low bile production in the first week after a liver transplant.^88,89^ However, MDR3 recovers at a slower rate than BSEP, resulting in a higher bile salt-to-phospholipid ratio during the first days after liver transplantation, which is aggravated by longer ischemia times.^90^ Phospholipids detoxify hydrophobic bile salts by the formation of micelles; thus, when bile salts outweigh phospholipids, the cytotoxic hydrophobic bile salts can cause increased damage to cholangiocytes. A high ratio has been shown to be associated with more severe injury to the bile ducts and with the development of post-transplant cholangiopathies.^88^ How these findings apply to the perinatal and neonatal bile duct following the ischemia and reperfusion of birth has not been examined, and although multiple studies have shown marked changes in the composition of the bile acid pool between the pre- and peri-natal periods,^91-93^ it is not clear what the exact bile acid-to-phospholipid ratio is at either time, whether bile is more toxic after birth, and which birth-related factors specifically influence bile composition. An increase in hydrophobic bile salts after birth would likely be more damaging in the neonate than in the adult because neonatal cholangiocytes lack a protective glycocalyx, as described above, but this requires further investigation.

Thus, the fetal and neonatal EHBD differ in important ways that render them susceptible to injury. In particular, they are highly susceptible to oxidative stress, and initial cholangiocyte damage in the context of an immature epithelial layer and submucosa may lead to bile leakage and extensive submucosal and basal epithelial damage. Overwhelming ROS production and irreversible injury to the duct could become fulminant after birth, when this oxidative event may lead to increased cell damage and death and, potentially, increased bile salt toxicity.

## In Utero Repair

### Fetal Vs Adult Wound Healing

Wound healing refers to the restorative response of a tissue to any type of damage. Generally, this response follows a sequence beginning with inflammation, then cellular proliferation and tissue repair, and finally, tissue remodeling. In some cases, wound healing is regenerative, meaning that the tissue is restored to its original state, while in other cases it is reparative, meaning that the function of the tissue has been mostly restored, although structural abnormalities (e.g., scar tissue) remain. In at least some tissues of the early and mid-gestational fetus, these typical wound healing stages result in full regeneration of the damaged tissue—a process termed fetal wound healing, described previously for skin, heart, lung, and tendon^94-97^ and more recently for the EHBD.^33^ In contrast, in the postnatal wound, wound healing typically results in a collagen-rich scar with reduced tensile strength. This postnatal process of so-called adult wound healing can also degenerate into fibrosis, with chronic inflammation, myofibroblast accumulation, and aberrant and excessive collagen type 1 deposition. The main features of fetal vs. adult wound healing are summarized in Table 1. Fetal wound healing has been extensively studied in the skin and much of the information available is derived from experiments in models of skin injury. Recovery from injury in other fetal organ systems has been less-well studied.

**Table 1.** Features of Fetal and Adult Wound Healing.

Fetal wound healing	Adult wound healing
Extracellular matrix	
• Marked increase in HA after injury^98^ • Prolonged increased HA, up to 3 weeks after initial injury^98^ • Predominantly high molecular weight HA; low levels of degradation^99,100^ • High expression of HA receptors and HA synthases.^101,102^ • Collagen rapidly deposited^95,103^ • Collagen fibers finely organized in reticular or basket-weave pattern^104-106^ • High collagen 3-to-collagen 1 ratio^107^ • Collagen bundles minimally crosslinked^103^ • High MMP-to-TIMP ratio^108,109^	• Slightly increased HA levels up to 3 days after initial injury^110^ • Low expression of HA receptors and HA synthases^101,102^ • Predominantly low molecular weight HA; high levels of degradation^99,110^ • Collagen deposition delayed^103^ • Collagen fibers densely packed in thick parallel bundles^111,104^• Low collagen 3-to-collagen 1 ratio^107,112^ • Collagen bundles highly crosslinked^103^ • Low MMP-to-TIMP ratio^109,113^
Cellular and soluble components	
• Elevated baseline levels of HIF1α, no upregulation in wound healing^114^ • High levels of anti-inflammatory IL-10^115^ • Low TGFβ1, high TGFβ3^116^ • Fibroblasts: high matrix synthesis but almost no myofibroblasts^111^ • High numbers of mesenchymal stem cells at site of injury^117^ • Attenuated inflammatory response^107^ - Decreased neutrophil migration - Absence of macrophages - Immature mast cells	• No baseline levels of HIF1α, but increased levels in wound healing^114^ • High levels of pro-inflammatory IL-6 and IL-8^115,118^ • High TGFβ1, low TGFβ3^116^ • Fibroblasts: low matrix synthesis and myofibroblasts present throughout wound healing^111^ • Low numbers of mesenchymal stem cells at site of injury^119^ • Strong inflammatory response^107^

Abbreviations: HA, hyaluronic acid; HIF1α, hypoxia-inducible factor α; IL, interleukin; MMP, metalloproteinases; TGFβ, transforming growth factor β; TIMP, tissue inhibitors of metalloproteinases.

The transitional period from a fetal to an adult-type of wound healing program occurs around 24 weeks of gestation in humans, at least for skin.^120^ In rodents, this transition for skin takes place between gestational days 17 and 19^121,122^ and in sheep between gestational days 100 and 120.^95^ During this period, wound healing can be highly variable and demonstrate elements of both fetal and adult wound healing (Table 1), although this may differ between skin and other organs.^123-125^

In the context of BA, initial damage to the EHBD may occur during the fetal wound healing phase and persist throughout the transitional phase when the ability to form scar tissue develops. Interestingly, we observed features of both fetal and adult wound healing in BA remnants,^32^ suggesting that both programs are part of BA pathophysiology.

### Fetal Wound Healing in Endoderm-Derived Organs

Mouse explant models have provided data on scar formation in heart and lung during embryology, showing that both heal scarlessly up to E18. The transition period between fetal and adult wound healing programs for these murine organs was determined to be 18–22 days gestation, similar to the skin.^126,127^

Studies in stomach and intestine, both of which, like the EHBD, are derived from endoderm (foregut and midgut, respectively), may have more direct relevance to the fetal bile ducts than data derived from skin research. Full thickness incisional gastric wounds in fetal sheep at a gestational age of 60 days, when the fetal skin heals scarlessly, showed pronounced scar formation.^123^ Another study yielded similar findings: a full thickness enterotomy on rabbit fetuses at a gestational age of 24 days (term 31 days), a period in which rabbit skin heals without a scar, resulted in intestinal healing with scar formation.^124^ Whether regenerative healing in the stomach and intestine occurs earlier in gestation is not known, although a study on chick embryos from 1981 may provide some insight. In this study, surgically induced intestinal perforations performed at day 9 (of a total of 21 days of incubation) resulted in stenosis or atresia (consistent with scarring repair) in almost 50% of chicks at hatching,^128^ while, in contrast, intestinal vascular lesions healed scarlessly in 100% of the embryos. Notably, in another study, the same authors found that induction of vascular lesions in late- (as opposed to early) gestation embryos resulted in most cases in intestinal atresia or stenosis.^129^ These observations suggest that both the embryological stage during which an injury is suffered and the type of tissue determine whether restorative or reparative wound healing occurs. They also suggest that the type of injury influences the nature of the wound healing program, which is particularly interesting in the context of BA given that it may result from a variety of different kinds of injuries. It is possible that viral infections, toxins and vascular events each have specific windows during gestation when EHBD damage could lead to scarring and BA. More work is needed to study fetal wound healing and injury in the EHBD.

### Collagen in Fetal Wound Healing: Type 1 Collagen Vs Type 3 Collagen

Fetal wound healing as a general process lacks the excessive collagen type 1 deposition that is typical of adult wound healing, although ECM remodeling still occurs. Notably, the ratio of pro-regenerative collagen type 3 to pro-fibrotic collagen type 1 is higher in the fetus than in the adult. Collagen type 3 fibers are smaller than type 1 fibers, promote a reticular deposition of fibers and reduce fibroblast differentiation into myofibroblasts—all of which may reduce scarring.^130-132^ Crosslinking of type 1 collagen, which is associated with increased matrix rigidity and scar formation, is low early in development.^103^ Additionally, TGFβ1, which is dominant in adult wound healing, prevents collagen degradation and enhances replacement of type 3 collagen by type 1^131^; not surprisingly, collagen degradation by matrix metalloproteinases (MMPs) occurs more rapidly and to a greater extent in fetal wounds compared to adult-type wounds.^108^ Thus, of the 2 major fibrillar collagens in the EHBD, type 3 collagen may contribute to a regenerative environment in the fetus whereas increased type 1 collagen in the older EHBD leads to increasing stiffness, which enhances the development of fibrosis.

### Hyaluronic Acid in Fetal Wound Healing: Friend or Foe?

**Figure 6.**
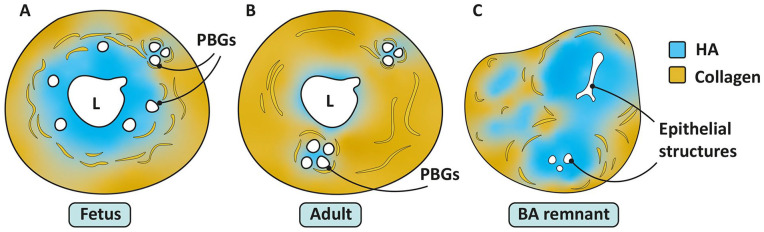
Relative proportions and organization of HA and collagen in the fetal (A) and adult (B) EHBD and BA remnant (C). Note the clustering of PBGs in (A) and (B) that coincides with local collagen replacement. Based on ref. de Jong et al.^32^ Abbreviations: BA, biliary atresia; EHBD, extrahepatic bile duct; HA, hyaluronic acid; PBGs, peribiliary glands.

HA is a glycosaminoglycan made up of widely variable numbers of repeating dimers of D-glucuronic acid and *N*-acetyl-D-glucosamine and is found at high levels in the ECM of many tissues, including skin and EHBD. In contrast to the ECM of adult skin and EHBD, in which type 1 collagen predominates, HA appears to be the dominant matrix component in the fetus.^5^ We recently reported that the EHBD wall of the normal fetus is composed of a wide layer of HA located directly around the lumen, with collagen at the periphery.^5,32^ During gestation, this HA layer is gradually replaced by collagen deposited from the periphery inward (Figures 3(b) and 6(a and b)). HA is highly negatively charged, resulting in repulsion between HA polymers that “opens up” the subcellular space and thereby creates a permissive environment for epithelial structures (such as peribiliary glands) to migrate and expand and for inflammatory cells to infiltrate the wound bed during wound healing. The large negative charge also contributes to the ability of HA to attract and retain water, resulting in a swelling capacity of 10,000 times its own volume.^133^

HA is synthesized in high molecular weight (HMW) form (>1.5 MDa) and is degraded to fragments with a low molecular weight (LMW) (<250 kDa); the average size of HA in tissues is determined by the balance of synthesis and degradation. Hyaluronidases 1 and 2 (HYAL1 and HYAL2) are the main enzymes that break the long HA polymers into shorter fragments. Free radicals and reactive oxygen species (ROS) are also known to have high HA degradation potency by causing direct fragmentation (rather than enzyme-mediated degradation).^134,135^

HMW and LMW HA have distinct biological effects on cells. HMW HA promotes an anti-inflammatory and pro-proliferative environment and suppresses angiogenesis,^136^ whereas LMW HA fragments induce angiogenesis and inflammation.^137^ HMW HA polymers protect against lymphocyte-mediated cytolysis, favor formation of macrophages with immunosuppressive phenotypes, and reduce neutrophil and macrophage migration in the presence of lipopolysaccharide (LPS).^138-140^ In contrast, LMW HA can act as a damage-associated molecular pattern (DAMP) by interacting with TLR-2/-4 and inducing pro-inflammatory pathways.^141^ Generally, healthy solid tissues contain HA predominantly in HMW form, whereas high amounts of LMW HA are found in pathological conditions including adult tissue remodeling and tumors.^142^ The average size of HA is different between adult and fetal EHBDs and, at least in the mouse, is significantly smaller in the adult than in the fetus/neonate.^32^ In the fetus, HMW HA plays a prominent role in tissue development and delays differentiation.^133,143^ This contributes to an environment in fetal tissues that is primed for rapid cellular proliferation and migration.

In adult wound healing, HMW HA is produced following the initial injury but is broken down within days into smaller fragments, promoting inflammation and neovascularization. In the fetus, however, increased levels of HMW HA are present at the wound site for up to 3 weeks. Experimental degradation of HA in fetal skin wounds promoted “adult-like” scarring, indicating that HMW HA plays an important role in regenerative wound healing in the fetus.^98,99,110^ Similarly, we showed in neonatal rat EHBDs (an age at which the adult wound healing program is predominant), that degradation of HA increased after toxin-induced damage. This suggests that HA in the EHBD is degraded after postnatal damage, as per a typical adult-type wound healing response.^32^

Together, these data suggest that HA plays a critical role in fetal wound healing and that its biology has profound effects on surrounding structures and cells. In recently published work, we showed that hypoxic damage to fetal sheep EHBDs resulted in a significant expansion of the HA layer, together with increased bilirubin levels and intrahepatic cholestasis.^32^ BA remnants obtained at Kasai surgery had similarly increased levels of HA, organized around epithelial structures and thus resembling the ECM of the fetal EHBD (Figure 6).^32^ We hypothesized that swelling of the HA layer in the fetal EHBD plays a role in the pathophysiology of BA; we proposed that expansion and persistence of the HA layer after early gestational injury contributes to narrowing of the fetal EHBD lumen followed by increased HA degradation, inflammation, and collagen deposition late in gestation and at birth when the transition to an adult wound healing program takes place.^32^

### Cellular and Soluble Components in Fetal Wound Healing

Fetal skin fibroblasts behave very differently than adult fibroblasts. Unlike adult fibroblasts, fetal fibroblasts do not differentiate into myofibroblasts—this ability slowly develops during gestation.^111^ These cells are also able to simultaneously synthesize collagen and proliferate, in contrast to adult fibroblasts, which decrease collagen synthesis while proliferating.^111^ Fetal skin fibroblasts are highly active and migratory and rapidly produce matrix^144^; this, in combination with their low contractility (as well as propensity to produce type 3 collagen rather than type 1), leads to a loose, soft, and “malleable” collagen matrix in fetal tissues and wounds. This fetal matrix, in contrast to the rigid and contractile matrix deposited as part of the adult wound healing program, facilitates the movement of cells that is necessary for rapid regenerative healing.^107^ Mouse neonatal EHBD fibroblasts have high proliferation rates and increased ECM production compared to adult fibroblasts,^5,32^ suggesting that the findings detailed above for skin may be relevant to the EHBD. Differences in fetal and adult fibroblast populations in the EHBD are not well understood, however, and more studies are needed before we can reach any conclusions about their relevance to the mechanism of BA.

Fetal tissues show reduced immune cell recruitment and activation after damage, contributing to lower levels of the pro-inflammatory interleukins (IL)-6 and -8. Instead, fetal tissue and amniotic fluid is rich in the anti-inflammatory cytokine IL-10.^145^ In IL-10 knock-out mouse fetuses, skin wounds healed with increased inflammation and scarring compared to controls^115^; adult wounds that overexpressed IL-10, in turn, showed reduced inflammation and scarless healing.^146^ This suggests that IL-10 plays an important role in fetal wound healing. Moreover, IL-10 upregulates hyaluronic acid (HA) synthases (HAS) 1, 2, and 3 and stimulates fetal fibroblasts to produce an HA-rich matrix.^147,148^ Multiple studies have found an association between BA and increased serum levels and differential expression of IL-10.^149-152^ In BA, increased IL-10 levels at the time of Kasai surgery may be the residua of fetal wound healing from the time of initial damage, although it appears that the restorative effect of IL-10 is not able to outweigh the subsequent fibrosing and inflammatory response in these patients.

## Putting It All Together

### The In Utero Biliary Damage-Repair Response Leading to BA

**Figure 7.**
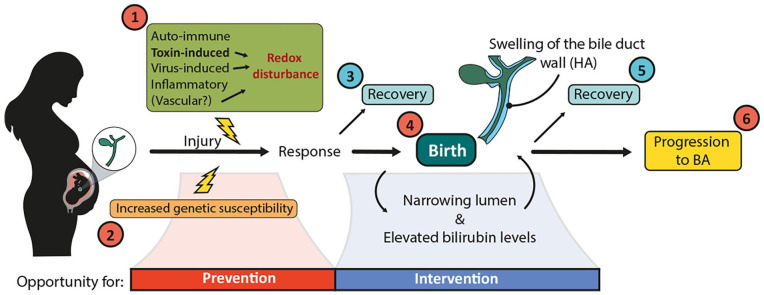
Proposed model to explain the pathophysiology of BA. Numbers in red indicate potential factors/timepoints that contribute to the progression of BA. Numbers in blue indicate timepoints when a portion of fetuses/neonates are hypothesized to recover from initial damage to the EHBD. This hypothesized scenario includes (1) several etiologies that could lead to damage to the EHBD at certain timepoints in gestation. The severity of the insult, timepoint in gestation and (2) genetic susceptibilities likely determine the response of the EHBD. Depending on the response, the duct could undergo regenerative healing in utero (3) or potentially HA deposition with duct swelling. Intrapartum ischemia-reperfusion, which leads to increased oxidative stress, may further fuel a wound healing response of the EHBD (4), including swelling of the HA layer, causing an increasingly narrowed lumen and elevated bilirubin levels; however, this response may be transient in a portion of newborns, resulting in recovery of the duct (5), while a small number of newborns are unable to recover and progress to clinical BA (6). Abbreviation: BA, biliary atresia.

We propose a new model of BA based in part on the data discussed here (Figure 7). First, we suggest that multiple environmental insults can lead to prenatal bile duct damage and that redox perturbations are a common feature among many of them. The severity of the insult, timepoint in gestation and genetic susceptibilities are likely to determine the response of the EHBD, such that mild damage may lead to duct recovery, with a program of fetal wound healing resulting in complete duct regeneration. Severe damage and ongoing redox imbalances, on the other hand, may prime the duct for progression of injury in response to the severe oxidative stress that occurs during birth. Some ducts may still be able to heal at this point; however, others may demonstrate progressive scarring and ultimately irreversible fibrosis, with the development of frank BA.

The EHBD demonstrates a program of typical fetal wound healing (with high levels of HA and low levels of collagen deposition) at least early in gestation, after which it gradually converts to a more collagen-dominant, adult-type response. The implications of HA deposition circumferentially around the duct are not clear and may vary depending on the stage of gestation. We propose that HA deposition and swelling may cause narrowing of the lumen and elevated conjugated/direct bilirubin levels. The healing response associated with HA may lead to duct repair and only cause transient narrowing of the lumen, with elevated bilirubin levels that return to normal within days to weeks, in line with the clinical course of several animal models of BA.^78,79,153^ In some settings, however, particularly late in gestation and after birth, duct narrowing and a combined fetal/adult wound healing program may lead to liver damage or be associated with duct scarring and progression to a BA phenotype. These scenarios raise the possibility that neonates diagnosed with BA represent the so-called “tip of the iceberg” and that there is a significant group of fetal and neonatal EHBDs that undergo repair without further liver or bile duct sequellae.

Clinical evidence consistent with this hypothesis was provided recently by a large clinical study reported by Harpavat et al^2^ in 2020, for which the authors assessed the conjugated/direct bilirubin levels of 124,385 newborns within 60 hours of birth and then at day 14. Approximately 0.05% of newborns had persistently elevated direct/conjugated bilirubin levels for over 14 days after birth but were otherwise healthy, never developing BA or any other liver disease. While there are many reasons for elevated direct/conjugated bilirubin levels that are spurious or unrelated to obstructive cholestasis, we hypothesize that some of these newborns experienced transient damage to the EHBD with HA-mediated narrowing, but recovered before total obstruction occurred (see (5) in Figure 7). Investigating this hypothesis will have important implications for early intervention and potential therapy of BA.

**Figure 8.**
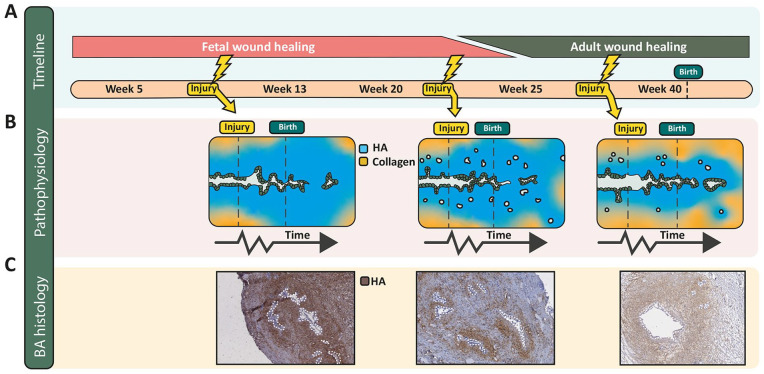
Wound healing programs during human gestation and proposed damage-repair response of the EHBD. (A) Transition from a fetal to an adult wound healing program around 25 weeks of human gestation, based on experiments in skin. It is unknown when features of fetal wound healing transition into adult-type wound healing in the EHBD. (B) Three potential timepoints of an initial EHBD injury, chosen to visualize the proposed different pathologies of the biliary epithelium and ECM seen at Kasai. The first damage-repair response (leftmost panel) is based on the absence of PBGs, an abundance of HA around the lumen, and a fetal wound healing program effective at the time of injury around 8/9 weeks of gestation. The middle panel is based on the high density and even distribution of PBGs throughout the submucosa and on features of the fetal wound healing program at 22 weeks of gestation. The rightmost panel, based on injury around 30 weeks of gestation, incorporates a thinning layer of HA around the lumen, clustered PBGs, and an at least partial adult wound healing program. These panels are simplified, but propose a novel perspective in which the EHBD phenotype resulting from an insult, as observed at the time of Kasai, is dependent on the time during development when the insult occurred. (C) HA staining of BA remnants that correspond with each of the morphologies depicted in (B). The white dotted lines outline epithelial structures.

In addition to the severity of the insult and genetic susceptibility factors, the time during gestation at which an insult occurs is likely key in determining whether EHBD injury results in scarring or recovery. The morphological, structural, and functional state of the bile duct, the degree of redox regulation, and the wound healing programs in play at that time will determine both the susceptibility and the response of the duct. Based on current knowledge, we predict that an EHBD removed at Kasai will have a different appearance depending on when during gestation the initial injury was suffered (Figure 8). The damage-repair response early in gestation features a fetal wound healing program, immature cells, the absence of PBGs, and a predominance of HA in the biliary submucosa, which would result in a BA remnant (at the time of Kasai; 1–3 months) with few epithelial structures and high levels of HA (Figure 8(b) and (c)), left). Between gestational weeks 13 and 24, PBGs are numerous and dispersed throughout the wide HA layer of the EHBD wall and a fetal wound healing program continues to predominate, with persistently high levels of HA that are subsequently broken down and replaced by collagen when the switch to adult wound healing occurs in the last trimester and after birth. At Kasai portoenterostomy, the cross-sectional remnant would likely show many epithelial structures dispersed throughout the section that are immediately surrounded by HA, with peripheral collagen (Figure 8(b) and (c)), middle). Late in gestation, the outer PBGs are clustered and encircled by collagen and the submucosa is largely composed of collagen. We would predict that injury elicits an adult wound healing program with transient HA elevations and with relative preservation of PBGs and the lumen at Kasai portoenterostomy (Figure 8(b) and (c), right).

**Figure 9.**
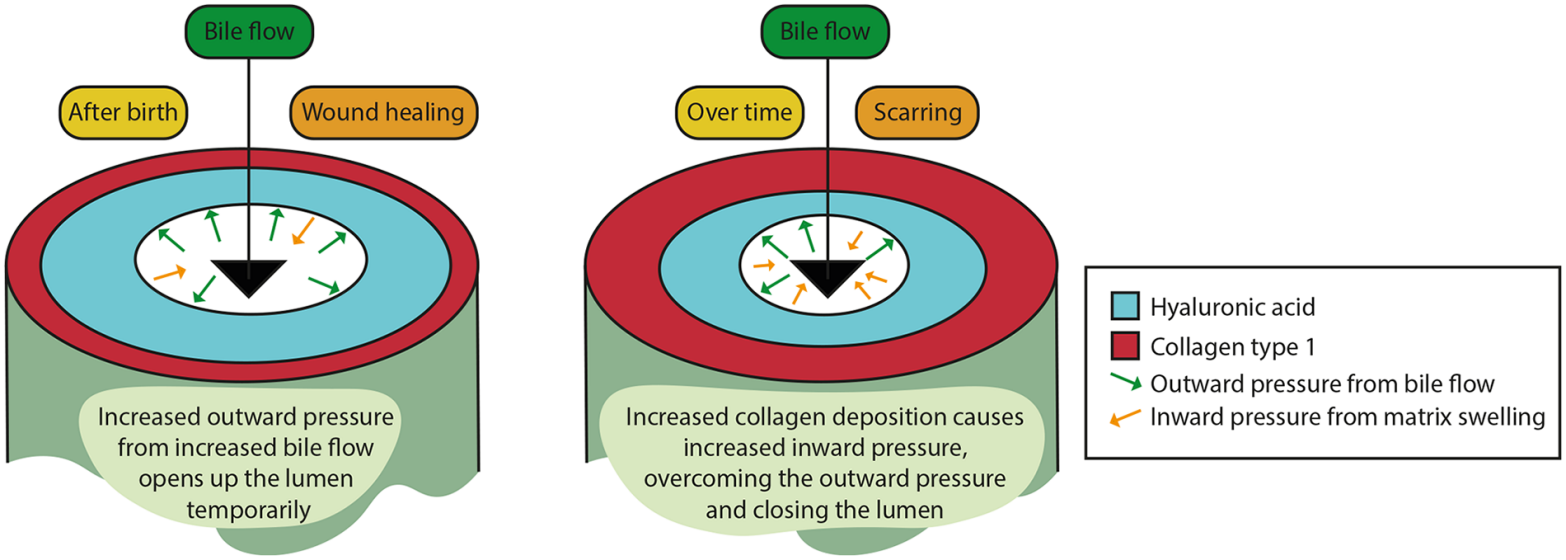
Hypothesis of the effect of increased bile flow and ECM remodeling on lumen diameter in BA pathophysiology. Bile flow increases after birth, while excess HA remains in the EHBD wall. The increased bile flow causes an increased intraluminal pressure, widening the lumen during this phase. Over time, collagen is increasingly deposited, and HA is only partly broken down, resulting in a rigid EHBD wall and swelling of the matrix. This causes an increased inward pressure, contributing to closure of the lumen, cholestasis, and elevated direct/conjugated bilirubin levels.

Collectively in all these scenarios, the fetal wound healing response remaining after birth results in increased HA deposition circumferentially around the epithelium and is then followed by increased collagen deposition (scarring). A bile duct wall composed primarily of HA with little collagen likely expands when intraluminal pressure increases via increased bile flow after birth. This may explain the variable direct bilirubin levels that can be observed^154,155^; however, when scarring occurs, the duct wall stiffens and the lumen progressively closes in spite of intraluminal pressure. Although this is a hypothesized scenario, histology is consistent with this sequence of events (Figures 8(c) and 9).

### Future Directions and Perspectives

In recent decades, little beyond incremental progress has been made in the prevention and treatment of BA. Despite extensive research, the standard therapy remains surgical: a Kasai portoenterostomy, followed (often years later) by a liver transplant.^156^ Moreover, we have yet to discover the etiologies leading to BA and the exact pathophysiology remains unclear. The mechanism we propose here highlights many critical points for further research, which will be essential for developing strategies to prevent and intervene in the progression of BA.

First, knowledge of normal development of the large bile ducts, in both humans and mice, remains incomplete. In particular, the following questions remain unanswered: how does the redox environment change during gestation in the large bile ducts, what are the most important redox couples at each timepoint in gestation, and how does redox state influence biliary development? In addition, it is unclear how the formation of the ductal plate is initiated, how the intrahepatic duct system and the extrahepatic duct system connect between gestational week 10 and 13, and how this differs between humans and mice. Importantly, it is currently unknown how wound healing evolves over time—that is, when does the wound healing program convert from a fetal-type to an adult-type, and what is the period of overlap? Additionally, is the outcome—repair or total obstruction—influenced by the time in gestation and nature of the injury?

Second, the role of birth in the progression of BA is yet unexplored. Studies performed in the context of perinatal injuries in general have revealed a tremendous impact of birth on redox state, but details of the redox state of the EHBD during and after birth are not known.^81,82,157^ Additionally, bile composition before and after birth should be investigated in more detail given that toxic bile could accelerate damage and contribute to the progression of BA. These studies could identify exciting new avenues for therapy of BA, given that many cellular processes are regulated by redox state and determinants of injury progression to BA vs recovery may be discovered.^43^

Third, pathways leading to recovery of the EHBD after perinatal damage should be defined. Currently available animal models of BA, which demonstrate that a small but reproducible percentage of animals undergo recovery,^78,79,153^ could be used to study cellular pathways that contribute to healing as opposed to progression of fibrosis. Categorizing progressing vs recovering animals soon after injury could identify an early stage of bile duct damage that is impossible to study in humans and, more importantly, might uncover factors contributing to recovery that potentially could lead to curative therapies.

Figure 7 suggests time frames during which prevention and curative therapy of BA may be possible. To prevent BA, there must be increased effort toward identification of genetic susceptibilities and environmental agents (toxins and viruses) that specifically target the fetal EHBD. Medical therapy for BA may seem far away; however, with recent advancements leading to earlier diagnosis, intervention may become feasible. Therapeutic intervention early after birth could promote recovery as opposed to fibrosis. Based on existing data, interesting options for intervention include NAC, to mitigate oxidative stress and prevent extensive cholangiocyte death; 4-methylumbelliferone to reduce deposition and thereby swelling of HA and to promote bile drainage^158^; and ursodeoxycholic acid, to mitigate oxidative stress and the formation of toxic bile and to promote bile drainage.

In conclusion, viewing BA from the perspective of the pre-, peri-, and postnatal damage-repair responses suggests exciting new research areas and possibilities for therapy.
